# The Cross-Species Mycobacterial Growth Inhibition Assay (MGIA) Project, 2010–2014

**DOI:** 10.1128/CVI.00142-17

**Published:** 2017-09-05

**Authors:** Michael J. Brennan, Rachel Tanner, Sheldon Morris, Thomas J. Scriba, Jacqueline M. Achkar, Andrea Zelmer, David A. Hokey, Angelo Izzo, Sally Sharpe, Ann Williams, Adam Penn-Nicholson, Mzwandile Erasmus, Elena Stylianou, Daniel F. Hoft, Helen McShane, Helen A. Fletcher

**Affiliations:** a Aeras, Rockville, Maryland, USA; b The Jenner Institute, University of Oxford, Oxford, United Kingdom; c Center for Biologics Evaluation and Research, Food and Drug Administration, Bethesda, Maryland, USA; d South African Tuberculosis Vaccine Initiative and Division of Immunology, Department of Pathology, University of Cape Town, Cape Town, South Africa; e Departments of Medicine, Microbiology, and Immunology, Albert Einstein College of Medicine, Bronx, New York, USA; f Department of Immunology and Infection, London School of Hygiene & Tropical Medicine, London, United Kingdom; g Colorado State University, Fort Collins, Colorado, USA; h Public Health England, Porton Down, Salisbury, United Kingdom; i Saint Louis University, St. Louis, Missouri, USA; UMKC School of Medicine

**Keywords:** mycobacterial growth inhibition assay, MGIA, tuberculosis, correlates of immunity, vaccines

## Abstract

The development of a functional biomarker assay in the tuberculosis (TB) field would be widely recognized as a major advance in efforts to develop and to test novel TB vaccine candidates efficiently. We present preliminary studies using mycobacterial growth inhibition assays (MGIAs) to detect Mycobacterium bovis BCG vaccine responses across species, and we extend this work to determine whether a standardized MGIA can be applied in characterizing new TB vaccines. The comparative MGIA studies reviewed here aimed to evaluate robustness, reproducibility, and ability to reflect *in vivo* responses. In doing so, they have laid the foundation for the development of a MGIA that can be standardized and potentially qualified. A major challenge ahead lies in better understanding the relationships between *in vivo* protection, *in vitro* growth inhibition, and the immune mechanisms involved. The final outcome would be a MGIA that could be used with confidence in TB vaccine trials. We summarize data arising from this project, present a strategy to meet the goals of developing a functional assay for TB vaccine testing, and describe some of the challenges encountered in performing and transferring such assays.

## INTRODUCTION

The tuberculosis (TB) vaccine field is severely hampered by the lack of defined immune parameters that correlate with vaccine efficacy in human studies. There has been recent progress, such as the finding that antigen-specific gamma interferon (IFN-γ) enzyme-linked immunosorbent spot assay (ELISpot) responses correlate with reduced risk of developing disease in Mycobacterium bovis BCG-vaccinated South African infants (1). However, heterogeneous BCG vaccine responses are observed and may be associated with the immune environment at the time of vaccination, including the proportions of monocytes and activated HLA-DR^+^ CD4^+^ T cells (1, 2). Development of a validated functional biological response assay capable of assessing the potential of a vaccine-induced immune response to protect against Mycobacterium tuberculosis infection or TB disease would represent a major advance in the effort to develop TB vaccines. Such an assay would circumvent the need to identify or fully understand the specific aspects of the immune response contributing to this effect. Mycobacterial growth inhibition assays (MGIAs) represent one such clinically relevant approach. Using whole blood or peripheral blood mononuclear cells (PBMCs), MGIAs offer an unbiased measure of the vaccine-induced antigen-specific immune response, taking into account the ability of this response to function in its respective immune environment. MGIAs also have the potential to decrease the time and resources required to evaluate a new TB vaccine in preclinical studies.

The MGIA project brought together an international group of scientific experts to work on growth inhibition assays from 2010 to 2014. Participants included researchers from regions in which TB is endemic, such as Cape Town, South Africa, and areas in which TB is not endemic, such as the United Kingdom and Washington, DC, St. Louis, MO, New York, and Colorado in the United States.

## GOALS OF THE MGIA PROJECT AND ASSAY SELECTION

The MGIA project was initially supported by a World Health Organization (WHO) working group and was viewed as the follow-up project to a whole-blood enzyme-linked immunosorbent assay (ELISA) that the WHO had recommended previously (3). The aim was to develop a functional assay that could be applied in all clinical trials of TB vaccine candidates, thus aiding the identification of correlates of immunity. Although several MGIAs had been described previously in the literature (recently reviewed in reference 4), little work had been done on qualifying an assay that could be transferred across laboratories, using a standardized reproducible method for vaccine assessment. This was a major goal of the project, which was funded by Aeras and the U.S. Food and Drug Administration (FDA). Prior to 2010, most MGIA development had focused on whole-blood systems and had shown promise (5–10). However, especially given the logistical challenges associated with real-time processing of fresh blood samples in clinical vaccine trials, efforts were made in this project to assess the potential of using cryopreserved PBMCs and to compare the outcomes with those from whole blood. The specific early goals were as follows: (i) to assess the variability, reproducibility, and transferability of four different MGIAs in naive, BCG-vaccinated, and BCG-revaccinated subjects; (ii) to compare PBMC and whole-blood MGIA responses induced by wild-type BCG and a new recombinant BCG vaccine; (iii) to determine the effect of M. tuberculosis infection status on inhibition of mycobacterial growth in a population where TB is endemic (South Africa); (iv) to test sera obtained from subjects enrolled in the enhanced BCG vaccine MGIA study for antibodies to capsular and cell surface constituents, including major polysaccharide antigens, and to compare the preimmunization and postimmunization antibody levels with MGIA outcomes; and (v) to explore other immune mechanisms underlying mycobacterial growth inhibition (e.g., by correlation with ELISpot and intracellular cytokine staining flow analyses of blood samples from the BCG study cohorts).

Although four previously reported MGIAs were considered for initial evaluation, a whole-blood assay based on the methods of Wallis et al. was selected as a focus for this project, using the Bactec mycobacteria growth indicator tube (MGIT) system to quantitate mycobacterial growth (5) (referred to here as the direct MGIA). The rationale for this decision included the relative simplicity of the assay and its use of standardized reagents and equipment that are readily available in many clinical TB laboratories. Furthermore, the assay had been used previously to demonstrate antimycobacterial activity of bactericidal drugs that correlated with sterilizing activity observed during *in vivo* therapy (5, 11). The Bactec MGIT system has several advantages over traditional CFU-based methods of mycobacterial quantification, as it utilizes oxygen depletion as a detection method that is sensitive not only to the number of viable bacteria but also to their rates of metabolism and growth. It is unaffected by clumping and does not require serial dilutions, providing an accurate computer-generated readout based on validated technology.

**FIG 1:**
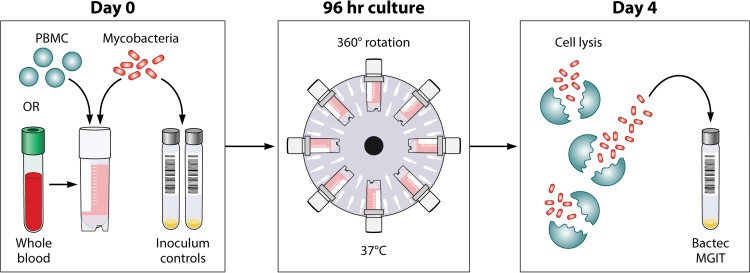
Schematic of the MGIT mycobacterial growth inhibition assay method. Whole blood or cell culture medium containing the appropriate concentration of PBMCs (or mouse splenocytes in mouse studies) is inoculated with an equal volume of mycobacteria at a low MOI (∼1 CFU/10,000 PBMCs). Cultures are incubated in 2-ml tubes at 37°C for 96 h, with 360° rotation. Cells are then lysed to remove red blood cells and/or to release intracellular mycobacteria. The lysate is inoculated into Bactec MGITs. The tubes are placed in the Bactec 960 system and incubated at 37°C until positivity is detected by fluorescence. On day 0, direct-to-MGIT viability controls are set up by directly inoculating MGIT tubes with the same volume of mycobacteria as the samples. The TTP data are converted to log_10_ CFU values using a standard curve, and the final values are expressed as absolute CFU values or values relative to the control value, indicating the amount of growth inhibition that occurred during the culture period.

It was also agreed that an adaptation of this assay using cryopreserved PBMCs should be developed and evaluated for comparison with whole-blood results. It is widely accepted that the cellular immune response is central to protection against M. tuberculosis and, although innate whole-blood components such as neutrophils are known to play a role in the host response, they may be less relevant for vaccine-induced effects on adaptive immunity. Furthermore, the use of cryopreserved cells could aid in transferability of the assay to different trial sites, eliminating the need to evaluate samples in real time using fresh material. Rather, cells could be stored and analyzed in the future, which not only is logistically simpler but also would negate the need for a Bactec MGIT system at every site. Finally, the ability to use cryopreserved cells would enable additional retrospective studies of samples from historical clinical vaccine trials, for validation and exploratory work. A schematic diagram and a description of the method for the direct MGIA are provided in Fig. 1.

The studies involved in this project are presented chronologically; work began using samples from clinical studies (as the ultimate goal was development of a human assay), but the challenges encountered resulted in a decision to focus on preclinical adaptations in the latter stages. One of the greatest impediments to initial MGIA developmental efforts using human samples was the lack of availability of sufficiently large single whole-blood or PBMC samples from recently BCG-vaccinated volunteers; this was required for assay optimization and assessment of reproducibility between laboratories. Using animal samples such as mouse splenocytes, however, it is possible to overcome this issue, as 50 million to 100 million splenocytes may be isolated from each mouse. Furthermore, the ability to select age- and sex-matched naive inbred animals removes a number of potential sources of variability found in human clinical trials. Animal models also provide the opportunity for biological validation by correlating assay outcomes with protection from *in vivo* challenges with pathogenic M. tuberculosis (see below).

Participants in the BCG vaccination and revaccination study were recruited under a protocol approved by the Oxfordshire Research Ethics Committee. Written informed consent was obtained from all individuals prior to enrollment in the trial. Human studies performed at the South African Tuberculosis Vaccine Initiative (SATVI) were approved by the Human Research Ethics Committee of the University of Cape Town. All adult participants provided written informed consent prior to participation in the studies. In the case of children, written informed consent was provided by a parent or legal guardian. All of the work at Saint Louis University was approved by the Saint Louis University Institutional Review Board, and PBMCs from independent healthy BCG-vaccinated subjects were collected under a protocol approved by the Institutional Review Board of the Albert Einstein College of Medicine. All animal studies were approved by the appropriate institutions, which used IACUC-approved protocols and methods. At Public Health England, all animal procedures and the study design were approved by the Public Health England, Porton Down, Ethical Review Committee and were authorized under an appropriate U.K. Home Office project license.

## EARLY OPTIMIZATION WORK

**FIG 2:**
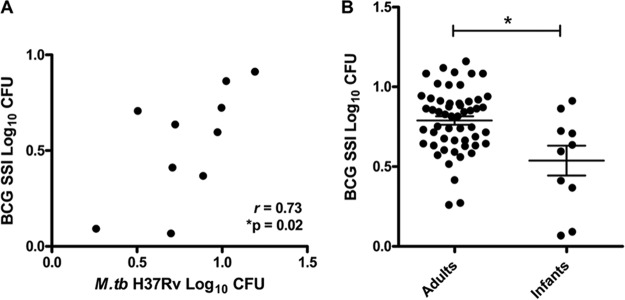
Direct whole-blood MGIA for South African infants. (A) Whole-blood samples from 10 South African infants were assessed using the direct whole-blood MGIA. There was a significant correlation between growth of BCG SSI and M. tuberculosis (*M.tb*) H37Rv (Spearman's *r* = 0.73; *P* = 0.02). (B) Whole-blood samples from 10 South African infants were compared with those from 53 South African adults using the direct whole-blood MGIA. There was a significant reduction in growth of BCG SSI in infants, compared with adults. *, *P* < 0.05, Mann-Whitney U test between groups.

BCG was selected as a surrogate for M. tuberculosis in the majority of these studies to avoid the need for biosafety level 3 (BSL3) facilities. It was established previously that measures of growth inhibition of BCG correlated with those of M. tuberculosis H37Rv in a murine MGIA (12), and this was confirmed in the direct MGIA (Fig. 2A). Early experiments using the direct PBMC MGIA indicated high intra-assay variability between replicate cultures. This was addressed by considering various aspects of assay preparation, the culture period, and 96-hour processing. It was determined that the standard antibiotic supplement (penicillin and streptomycin) in the culture medium should not be used at any stage of sample processing after thawing, due to the effect of artificially reducing the inoculum (13). However, this altered the multiplicity of infection (MOI), such that the vaccine effect was overwhelmed. A smaller inoculum resulted in increased intra-assay variability. To maintain the same initial inoculum volume, a lower MOI was achieved by using greater cell concentrations; although this did improve reproducibility, a balance had to be reached, given the limiting factor of cell numbers in many clinical trials. Experiments altering the MOI in the direct splenocyte MGIA have been described (14). Once the optimal MOI was identified, variability in the inoculum itself was addressed by comparing different declumping methods, such as vortex-mixing, filtering, and sonicating. Vortex-mixing with glass beads resulted in low intra-assay variability while retaining high recovery of CFU. Other variables examined included BCG strain (14), culture rotation methods, and lysis agents. Variability between sites in whole-blood MGIA performance has yet to be defined but could result from factors such as differences in blood anticoagulants and inoculum strains.

## HUMAN MGIA STUDIES

### BCG vaccination and revaccination.

A clinical trial of BCG vaccination and revaccination in U.K. adults was conducted at the University of Oxford (13). The direct PBMC MGIA detected a significant improvement in mycobacterial growth inhibition following primary BCG vaccination, consistent with epidemiological data on the efficacy of BCG in this population. No improvement in mycobacterial growth inhibition was observed following BCG revaccination, using either the whole-blood or PBMC assay (13). This is in contrast to a previous study with U.S. volunteers, in which the direct whole-blood MGIA, as well as the primary lymphocyte MGIA (effector lymphocytes added to infected autologous monocytes [9]) and the secondary lymphocyte MGIA (using antigen-expanded T cells [6]), detected enhanced mycobacterial growth inhibition after BCG revaccination (7).

In the Oxford study, the direct PBMC MGIA demonstrated a stronger primary vaccine effect and greater reproducibility over repeated baseline bleeds, compared with whole blood (13), most likely due to the processing of longitudinal PBMC samples in one batch. This is not an option for fresh blood, which may suffer from batch effects, including those associated with different vials of mycobacterial inoculum. However, this was not the case in other studies, such as the AERAS-422 vaccine trial at Saint Louis University described below, in which the direct whole-blood MGIA proved less variable among subjects included in each vaccinated group, compared to primary, secondary, or titrated PBMC MGIAs (15).

An additional study was conducted in South Africa, comparing 10-week-old infants who were BCG vaccinated at birth with older adults. Infants demonstrated enhanced ability to inhibit growth of BCG SSI (but not M. tuberculosis H37Rv) in the direct whole-blood MGIA, compared with adults (Fig. 2B). These data suggest that BCG vaccination may induce preferential growth inhibition of BCG even in newborns.

### Study of immune correlates of risk.

Recently, Fletcher et al. reported the findings of a study on the immune correlates of risk among BCG-vaccinated infants in South Africa (1). In that population, BCG-specific IFN-γ ELISpot responses and levels of Ag85A-specific IgG antibodies measured at 4 to 6 months of age were associated with reduced risk of developing TB disease over the next 3 years of life (1). However, the ability to control mycobacterial growth in the direct PBMC MGIA was not associated with reduced risk of TB disease. This could be due to the very low frequency of BCG-specific IFN-γ-secreting T cells (median of <50 spot-forming cells/1 million PBMCs) and the fact that small numbers of PBMCs were used in the assay (it has since been established that the direct MGIA is more robust when greater numbers of PBMCs are used). Furthermore, autologous serum was not used in the assay; therefore, the assay would not have measured the contribution of Ag85A-specific IgG, or any other antibody, to growth inhibition (1). The study was also performed prior to subsequent further optimization of the assay.

### Comparison of wild-type BCG and a new recombinant BCG vaccine.

Hoft et al. performed the direct whole-blood MGIA as part of a clinical trial of AERAS-422, a live recombinant BCG expressing perfringolysin and three M. tuberculosis antigens, namely, 85A, 85B, and Rv3407 (15). Between November 2010 and August 2011, 24 volunteers were enrolled (AERAS-422, high dose, *n* = 8; AERAS-422, low dose, *n* = 8; Tice BCG, *n* = 8). High-dose AERAS-422 vaccination induced Ag85A- and Ag85B-specific lymphoproliferative responses and marked antimycobacterial activity detectable in the direct whole-blood MGIA (15). A systems biology approach using modular analysis identified positive correlations between postvaccination T cell gene expression and MGIA activity and negative correlations between postvaccination monocyte gene expression modules and MGIA activity. Unfortunately, the development of varicella-zoster virus reactivations occurred in two of eight vaccine recipients given the high dose of AERAS-422, resulting in discontinuation of AERAS-422 clinical development. However, these results demonstrated that the direct whole-blood MGIA could detect vaccine-induced increases in antimycobacterial immune activity (15).

### Assessment of M. tuberculosis infection status.

As described, there was a significant correlation in outcomes using BCG and M. tuberculosis in the direct whole-blood MGIA. However, no difference in the magnitude of growth inhibition was detected between healthy M. tuberculosis-infected and noninfected subjects, using either mycobacterial strain, in a region of South Africa in which TB is endemic (R. Baguma and T. J. Scriba, unpublished observations). This was in contrast to a previous study in which levels of growth of luminescent mycobacteria were significantly lower in blood from tuberculin skin test (TST)-positive subjects, compared with TST-negative subjects, in a region in which TB is not endemic (10). The discordance between these findings might have resulted from the cross-sectional designs, in which infection with M. tuberculosis was defined using a single IFN-γ release assay or TST, and it is not known whether volunteers from either study were recently or historically exposed to M. tuberculosis. Furthermore, despite being QuantiFERON negative, the South African volunteers had readily detectable T cell responses to BCG stimulation, suggesting high levels of immunological sensitization to mycobacteria (likely due to childhood BCG vaccination and/or exposure to environmental mycobacteria).

## IMMUNE MECHANISMS OF GROWTH INHIBITION

One major goal of this project was to elucidate immune mechanisms underlying the mycobacterial growth inhibition observed. The influences of IFN-γ-producing T cells, antibodies, monocyte/lymphocyte (ML) ratios, and hemoglobin (Hb) were assessed.

### IFN-γ-producing T cells.

The magnitude of mycobacterial growth inhibition following BCG vaccination in the Oxford study did not correlate with the antigen-specific IFN-γ ELISpot response, and a boosted IFN-γ ELISpot response observed following BCG revaccination was not reflected in enhanced MGIA activity (13). Among the South African volunteers, although QuantiFERON-TB Gold-positive individuals had greater levels of ESAT-6- and CFP-10-specific IFN-γ-secreting T cells, this did not translate into improved MGIA responses. These findings are consistent with previous observations that *in vitro* mycobacterial growth inhibition does not correlate with IFN-γ production (7, 8).

### Antibodies.

Chen et al. demonstrated that IgG antibody responses to arabinomannan (AM), a mycobacterial envelope polysaccharide, increased significantly following BCG vaccination, using samples from the Oxford trial (16). Their studies suggested that such antibodies, particularly when targeting specific oligosaccharide epitopes, may play a functional role in mycobacterial growth inhibition. Phagocytosis and intracellular growth inhibition were significantly enhanced when BCG was opsonized with postvaccination sera, and the enhancements correlated with IgG titers to AM. Furthermore, increased phagolysosomal fusion in M. tuberculosis-infected macrophages was observed with postvaccination serum but not prevaccination serum, indicating that intracellular growth reduction was FcR mediated. Interestingly, the authors also found that the antibody response at 4 weeks after BCG vaccination correlated with mycobacterial growth inhibition in the direct PBMC MGIA (16). A functional role of antibodies to AM has since been further supported by murine immunization studies with AM conjugate vaccines (17). These data highlight the importance of assessing the humoral compartment in MGIAs and comparing PBMC- and whole-blood-based approaches to better assess the potential contributions of antibody-mediated responses.

### Monocyte/lymphocyte ratio.

In blood samples from healthy volunteers, a greater proportion of monocytes with respect to lymphocytes (higher ML ratio) is associated with increased mycobacterial growth and increased probability of a type I IFN transcriptional signature (18). Altering the ML ratio *in vitro* affects the control of mycobacterial growth, with either very high or very low ML ratios being associated with poorer control in the direct PBMC MGIA (18). This is consistent with the observations that profoundly altered ML ratios were associated with increased risk of developing TB disease among non-HIV-exposed infants, HIV-exposed uninfected infants, HIV-positive adults starting antiretroviral therapy, and postpartum women (2, 19–21). Accordingly, Hoft et al. showed that antimycobacterial immune activity correlated positively with T cell expression signatures and negatively with monocyte expression signatures in the AERAS-422 study (15). Further studies with animal models may better define the subpopulations of monocytes and lymphocytes important for mycobacterial growth inhibition.

### Hemoglobin.

Using samples from the Oxford BCG vaccination study, a positive correlation was observed between mean corpuscular Hb levels and mycobacterial growth in the direct whole-blood MGIA (22). Experimental addition of Hb or ferric iron to PBMCs from both humans and nonhuman primates (NHPs) resulted in increased mycobacterial growth, an effect reversed by addition of the iron chelator deferoxamine. Furthermore, expression of Hb-related genes correlated significantly with mycobacterial growth in whole blood and, to a lesser extent, in PBMCs (22). This association between Hb/iron levels and mycobacterial growth may in part explain differences in outcomes between whole-blood and PBMC MGIAs and should be taken into account when using these assays.

## ANIMAL MGIA STUDIES

Given that early clinical studies indicate the need for more investigations before a human PBMC MGIA can be qualified, MGIA development using animal models of TB represents an important ongoing strategy. In addition to the benefits regarding sample volume and reduced heterogeneity described above, animal models have the important advantage that the MGIA outcomes could be correlated with protection from *in vivo* challenges with pathogenic M. tuberculosis, which would then biologically validate the assay as being capable of assessing effective antimycobacterial immunity. Where there is a need to test vaccine candidates for antigen dose, adjuvant dose, or antigen-adjuvant combinations, MGIAs could save time, animals, and funds. As the direct MGIA does not depend on any species-specific immune reagents, it can be easily adapted for use across a range of animal species.

In an effort to improve intersite reproducibility, the animal phase of the MGIA project utilized a single shared batch of frozen mycobacteria. The BCG batch was prepared at Aeras and tested for viability and reproducibility prior to distribution to project partners. Investigators also agreed that, for each experiment, the CFU of mycobacterial stocks would be determined and standard curves would be used to convert Bactec MGIT time-to-positivity (TTP) data to log_10_ CFU for the growth counts, for ease of data presentation and interpretation.

### Mouse model.

A MGIA using infected macrophages cultured separately from mouse splenocytes, and then combined, previously demonstrated reproducible growth inhibition data at 7 days of incubation (23). Using this MGIA, immune splenocytes from mice vaccinated with five different TB vaccines limited mycobacterial growth *in vitro*, compared to naive controls. Importantly, the vaccine-induced MGIA correlated at a group level with protective immune responses induced in experimentally matched animals, using a mouse model of pulmonary TB (23).

More recent studies have focused on simplifying the mouse MGIA model. Marsay et al. showed that the direct MGIA using splenocytes has the ability to detect mycobacterial growth inhibition following BCG vaccination (24). Furthermore, investigators from the Center for Biologics Evaluation and Research (CBER), FDA, used the direct splenocyte MGIA to demonstrate reproducible *in vitro* protective responses from mice immunized with either BCG or a subunit vaccine, and they showed that the responses correlated on a group level with *in vivo* protection data from experimentally matched mice (25). They also confirmed that mycobacterial quantification using the Bactec MGIT system correlated strongly with plating of organ homogenates and CFU counting and is faster and easier to perform than the traditional CFU method (26).

BCG gives a robust and reproducible protective effect against challenge with M. tuberculosis in C57BL/6 mice; therefore, this model has been used for further optimization of the mouse direct MGIA. Zelmer et al. sought to enhance the sensitivity of the direct splenocyte MGIA and found that detection of vaccine-induced inhibition could be improved by decreasing the MOI (27). It was also shown that the capacity to detect mycobacterial growth inhibition in BCG-vaccinated mice was time sensitive, with growth inhibition being detected only at the peak of the BCG immune response (approximately 6 weeks in C57BL/6 mice) (14). As there is no amplification of the antigen-specific immune response in the direct MGIA, it is only the ability of the cells resident in the spleen at the time of harvest that can be assessed for antimycobacterial capacity (14). Since BCG is a live replicating mycobacterium, there can be variations in the development of the peak of the immune response and variations in the persistence of BCG vaccine in lungs and spleen; this can affect the ability to reproducibly select the optimal time point for measuring vaccine responses (28, 29). However, selecting the time point for the peak immune response may be a lesser challenge in assessing a subunit vaccine, and the ability of MGIAs to assess the effectiveness of subunit vaccines has shown early promise (23).

### Guinea pig model.

Investigators at Colorado State University and Public Health England are in the process of adapting the direct MGIA for use with a guinea pig model. Early indications are that PBMCs provide more robust information than does whole blood, and further studies with vaccine candidates to examine the utility of PBMC MGIAs in the guinea pig model are in progress.

### NHP model.

The direct whole-blood MGIA has been adapted for use with NHP samples at the University of Oxford, in collaboration with Sharpe and colleagues at Public Health England. In a study of seven rhesus macaques, the assay demonstrated strong enhancement of mycobacterial growth inhibition following BCG vaccination, which was still significant following correction for changes in hemoglobin levels (22). Work is currently ongoing to optimize the NHP direct PBMC MGIA and to correlate the outcomes with protection from *in vivo* BCG and M. tuberculosis challenges, on an individual animal basis. Reproduction of the MGIA in NHPs could help to inform human studies and to provide biological validation of the assay.

## CONCLUSIONS

The MGIA represents a functional assay for assessing TB vaccine activity, which has the potential to correlate with vaccine-induced immune protection. To date, the direct whole-blood MGIA has demonstrated the most consistent effects across a range of different clinical vaccine studies in humans. However, because this assay must be performed within hours after blood drawing, it requires laboratory infrastructure close to the clinical site, making it difficult to assess in many vaccine trials, particularly in countries in which TB is endemic. Cryopreserved PBMCs are thus the preferred specimen type for logistical reasons, and the direct PBMC MGIA is currently undergoing further optimization and harmonization across laboratories as part of the European Research Infrastructures for Poverty Related Diseases collaborative infrastructure program (H. McShane and R. Tanner, personal communication). Use of MGIAs in animal models of TB provides an opportunity for biological validation with respect to protection from pathogenic *in vivo* challenges and could be more time- and cost-effective. Animal MGIAs could also reduce the number of virulent M. tuberculosis challenge experiments required to identify promising vaccine candidates for progression to clinical trials (30).

The results presented indicate that the MGIA has promise as a functional assay for the assessment of candidate TB vaccines. It also provides a tractable model for investigating the mechanisms involved in antimycobacterial immunity. For example, findings from this project have highlighted the importance of both lymphoid and myeloid cell-mediated immunity and antibody-mediated immunity in these assays. It would be interesting to consider how exposure of an individual to other mycobacterial species or infections (such as helminths) would affect the ability to control mycobacterial growth *in vitro*, and work to this end is under way. The influence of regulatory immune mechanisms and suppressors, which would be represented in unbiased samples such as whole blood and PBMCs, should also be explored.

In this report, we have summarized the numerous accomplishments of this project. Many parameters of a standardized MGIA that can be routinely performed in laboratories with access to Bactec MGIT equipment have been established. However, MGIAs are technically demanding, and it is clear that further work is required, particularly to reduce the variability intrinsic to functional assays. Use of cryopreserved PBMCs remains a challenge, as does the goal of qualifying this assay for use in human studies in particular. Utilization of the direct MGIA in animal models (especially mice and NHPs) for assessment of new TB vaccines seems more valuable at this time. A number of explorations of MGIAs as functional TB tests should be performed in the future, and some of these goals are as follows: (i) to demonstrate that the direct PBMC MGIA meets requirements as a qualified assay; (ii) to determine whether there are significant differences when BCG or M. tuberculosis strains are used for *in vitro* infection; (iii) to evaluate differences when MGIAs are used in countries in which TB is endemic or not endemic; (iv) to determine whether MGIAs can be used in M. tuberculosis-infected populations; (v) to identify factors in PBMCs and whole blood that mediate and influence growth inhibition; (vi) to evaluate the performance of the PBMC MGIT assay in trials of novel TB vaccine candidates; (vii) to determine whether the test is reproducible at different sites; and (viii) to determine whether the direct whole-blood MGIA can be further adapted or refined for use in vaccine or TB studies. If MGIAs were validated and shown to correlate reproducibly and consistently with efficacy demonstrated in clinical trials of candidate vaccines for TB, they would hold great potential. Much as neutralizing antibodies are currently used to assess the efficacy of certain other vaccines, MGIA results could be applied for regulatory purposes, as a surrogate marker of efficacy.
